# A *POT1* Founder Variant Associated with Early Onset Recurrent Melanoma and Various Solid Malignancies

**DOI:** 10.3390/genes15030355

**Published:** 2024-03-13

**Authors:** Aasem Abu Shtaya, Inbal Kedar, Lily Bazak, Lina Basel-Salmon, Sarit Farage Barhom, Michal Naftali, Marina Eskin-Schwartz, Ohad S. Birk, Shirley Polager-Modan, Nitzan Keidar, Gili Reznick Levi, Zohar Levi, Tamar Yablonski-Peretz, Ahmad Mahamid, Ori Segol, Reut Matar, Yifat Bareli, Noy Azoulay, Yael Goldberg

**Affiliations:** 1 Recanati Genetics Institute, Rabin Medical Center—Beilinson Hospital, Petach Tikva 4941492, Israel; asemab@clalit.org.il (A.A.S.); inbalkd@clalit.org.il (I.K.); lilybazak@clalit.org.il (L.B.); linab@clalit.org.il (L.B.-S.); saritfa1@clalit.org.il (S.F.B.); matarre@clalit.org.il (R.M.); yifatba1@clalit.org.il (Y.B.); noyaz@clalit.org.il (N.A.); 2 Unit of Gastroenterology, Lady Davis Carmel Medical Center, Haifa 3436212, Israel; ori_segol@clalit.org.il; 3 Faculty of Medicine, Tel Aviv University, Tel Aviv 6997801, Israel; zoharl@clalit.org.il; 4 Felsenstein Medical Research Center, Petach Tikva 4920235, Israel; 5 Pediatric Genetic Unit, Schneider Children’s Medical Center of Israel, Petch Tikva 4920235, Israel; nitzankei@clalit.org.il; 6 Clalit Genomic Center, Petach Tikva 5252247, Israel; michalnaf@clalit.org.il; 7 Genetics Institute, Soroka University Medical Center, Beer Sheva 8410101, Israel; blmarinaa@clalit.org.il (M.E.-S.); obirk@bgu.ac.il (O.S.B.); 8 Faculty of Health Sciences, Ben Gurion University of the Negev, Beer Sheva 8410101, Israel; 9 Genetics Institute, Carmel Medical Center, Haifa 3436212, Israel; shirlymd@clalit.org.il; 10 Genetics Institute, Rambam Health Care Campus, Haifa 3525408, Israel; g_reznick@rmc.gov.il; 11 Division of Gastroenterology, Rabin Medical Center—Beilinson Hospital, Petach Tikva 4941492, Israel; 12 Oncology Institute, Hadassah Medical Center, Jerusalem 9112001, Israel; tamary@hadassah.org.il; 13 Faculty of Medicine, Hebrew University of Jerusalem, Jerusalem 9190500, Israel; 14 Department of Surgery B, Carmel Medical Center, Haifa 3436212, Israel; ahmadma1@clalit.org.il

**Keywords:** *POT1*, cancer, melanoma, desmoid, breast cancer, Ashkenazi

## Abstract

POT1 (Protection of Telomeres 1) is a key component of the six-membered shelterin complex that plays a critical role in telomere protection and length regulation. Germline variants in the *POT1* gene have been implicated in predisposition to cancer, primarily to melanoma and chronic lymphocytic leukemia (CLL). We report the identification of *POT1* p.(I78T), previously ranked with conflicting interpretations of pathogenicity, as a founder pathogenic variant among Ashkenazi Jews (AJs) and describe its unique clinical landscape. A directed database search was conducted for individuals referred for genetic counselling from 2018 to 2023. Demographic, clinical, genetic, and pathological data were collected and analyzed. Eleven carriers, 25 to 67 years old, from ten apparently unrelated families were identified. Carriers had a total of 30 primary malignancies (range 1–6); nine carriers (82%) had recurrent melanoma between the ages of 25 and 63 years, three carriers (27%) had desmoid tumors, three (27%) had papillary thyroid cancer (PTC), and five women (63% of female carriers) had breast cancer between the ages of 44 and 67 years. Additional tumors included CLL; sarcomas; endocrine tumors; prostate, urinary, and colorectal cancers; and colonic polyps. A review of a local exome database yielded an allelic frequency of the variant of 0.06% among all ethnicities and of 0.25% in AJs. A shared haplotype was found in all carriers tested. *POT1* p.(I78T) is a founder disease-causing variant associated with early-onset melanoma and additional various solid malignancies with a high tumor burden. We advocate testing for this variant in high-risk patients of AJ descent. The inclusion of *POT1* in germline panels for various types of cancer is warranted.

## 1. Introduction

Telomeres are regions composed of repetitive nucleotide sequences that are located at the ends of linear chromosomes. They are directly involved in protecting the chromosomal ends from enzymatic degradation and from accidental DNA recombination during cellular division. With each cellular division, telomeres shorten. This phenomenon plays a pivotal role in the aging process and cellular lifespan. The telomerase enzyme synthesizes new telomere repeats with each cycle to maintain telomere length and promote genomic stability [1].

The role of telomeric dysfunction in disease progression and cellular instability underscores the significance of maintaining telomeric health and the continuous attempts to develop telomerase-directed therapies [2]. 

Altered telomere length is associated with various pathologies. Accelerated telomeric shortening has been linked to the early onset of many age-associated ailments and to certain genetic disorders [3,4], including hematopoietic failure, some forms of ectodermal dysplasia, aplastic anemia, pulmonary fibrosis, liver disease, and premature biological aging [5]. 

Telomeric dysfunction has also been shown to be directly connected to cancer development. Individuals with shorter telomeres were found to be at increased risk of multiple tumors such as lung, bladder, and various gastrointestinal malignancies [6]. Research has also shown that cancer cells often evade cellular apoptosis by employing mechanisms to counteract telomeric shortening; some do so by the activation of telomerase, leading to the addition of repetitive DNA sequences to chromosomal ends and sustaining telomeric elongation to enable cancer cells to undergo continuous divisions [6].

The POT1 protein is an essential subunit of the shelterin telomere binding complex. It directly binds to single-stranded telomeric DNA, protecting chromosomal ends from an inappropriate DNA damage response, and plays a role in telomere length regulation. Shelterin plays a vital role in telomere function by remodeling telomeric DNA into a protected structure and managing the regulation of telomere length. Loss or malfunction of shelterin proteins results in uncapped telomeres, which induce genomic instability. Lately, increasing evidence shows that long telomeres caused by variants in shelterin components (*POT1*, *TPP*1, and *RAP1*) also display an increased risk of cancer [7]. 

POT1 is involved in the regulation of telomerase-dependent elongation of the telomere. Some studies have suggested that it increases the processivity of telomerase, whereas others have identified a role of POT1 in the negative regulation of telomerase catalytic activity [8,9]. *POT1* is composed of three oligosaccharide/oligonucleotide (OB) fold domains, namely, OB1, OB2, and OB3. It binds to the telomeric single-stranded repeat via the OB1 and OB2 fold domains, while the OB3 fold domain enables its association with the TPP1 protein [10]. Variants in the OB1 and OB2 domains affect the single-stranded DNA-binding ability of *POT1*, while variants in OB3 diminish POT1’s affinity for TPP1, affecting its recruitment to telomeres [11]. 

Somatic *POT1* variants were initially identified in CLL cells [12]. Germline biallelic *POT1* variants have been reported in an infant with Coats plus syndrome [13]. Germline mono-allelic variants have been identified in various types of cancer in small series [12]. Cutaneous melanoma is the most commonly reported malignancy in POT1 carriers. Previous publications have described *POT1* variant carriers to be at increased risk of CLL, uterine cancer, lung cancer, sarcoma, and colon cancer. Functional studies have shown significant disruption of the protein in carriers [14,15,16]. Most such reports were limited by the small number of carriers. Lately, *POT1* variants associated with long telomere length were also shown to confer predisposition to a familial clonal hematopoiesis syndrome that is associated with a range of benign and malignant solid neoplasms [17].

The human *POT1* gene is comprised of 22 exons. The (NM_015450.3) c.233T>C p.(Ile78Thr) variant is located in exon 7 within the OB1 domain. The variant has been submitted to ClinVar by several clinical testing laboratories (variation ID 475073) with conflicting interpretations of pathogenicity, including uncertain significance and likely pathogenic, supported by one star. A study published in 2019 by Wong et al. highlighted the potential pathogenicity of the p.(I78T) variant within families showing a predisposition to melanoma [18]. In recent years, further research, functional assessments, and clinical data led to more submissions in ClinVar, claiming its pathogenicity and changing the variant classification to likely pathogenic according to the American College of Medical Genetics and Genomics (ACMG) guidelines.

In this study, we review the previous allusions of the *POT1* Ile78Thr variant, describe ten apparently unrelated carrier families, estimate its prevalence among AJs, and describe the clinical landscape among patients and families. This report could help to provide clinical data to design surveillance protocols and raise awareness to *POT1* as a cancer predisposition gene. We advocate testing the p.(I78T) variant in high-risk patients of AJ descent and including *POT1* in germline panels for various types of tumors.

## 2. Patients and Methods

This cohort included eleven index patients found by genotyping to have the p.(I78T), (NM_015450.3) c.233T>C, chromosome 7, g.124870933A>G, (GRCh38) germline variant in the *POT1* gene. Ten patients had been referred for oncogenetic counseling due to personal history of cancer; one was referred for trio-exome sequencing because of a developmental delay in her child. The relevant demographic, clinical, pathologic, and genetic data were retrieved from the medical files. 

Informed consent was obtained from all subjects involved in the study. The study was approved by the institutional review boards of all participating medical centers (approval no. 0847-22). 

Testing was performed via one of three genetic institutes in Israel between 2018 and 2023. It consisted of either next-generation sequencing with multicancer panels (Invitae Multi-Cancer Panel, Invitae CORP, San Francisco, CA; Blueprint Comprehensive Hereditary Cancer Panel, Blueprint Genetics, Espoo, Finland; and Clalit Genomic Center Hereditary Cancer Panel, Clalit Genomic Center, Petach Tikva, Israel) and/or whole exome sequencing in the CeGat laboratory (CeGaT GmbH, Tübingen, Germany). 

The genomic DNA obtained from the submitted samples was enriched for the targeted regions using a hybridization-based protocol and sequenced using Illumina technology. Sequencing libraries were size-selected with the bead-based method to ensure optimal template size and amplified by polymerase chain reaction (PCR). Regions of interest (exons and intronic targets) were targeted using the hybridization-based target capture method. The resulting sequences were aligned against the human genome build GRCh37/UCSC hg19 and analyzed for sequence variants. The different laboratories used various methods for the identification of sequence changes (variants) in the aligned reads. All labs had processes in place for detecting copy number variations (CNV). The clinical interpretation of identified variants was based on established guidelines, considering databases such as gnomAD, ClinVar, and HGMD for variant assessment. Output files containing the sequenced data and variant information were produced. The full list of genes included, sequencing methods in each panel, and the genotyping technique of exome sequencing are explained thoroughly in Supplementary File S1.

Haplotype analysis was completed by genetic testing for the microsatellite analysis. It was performed by using fluorescent PCR for size discrimination with capillary electrophoresis. Testing was performed on DNA extracted from the peripheral blood cells of six Ashkenazi individuals from different families. The PCR was performed in a 25 μL vessel containing 50% Taq Mix Red (PCR Biosystems Ltd., London, UK) and 5% dimethyl sulfoxide (DMSO) (Sigma Aldrich, St. Louis, MI, USA), using 0.1 μM of each primer. The reaction was thermocycled for 35 cycles using a program of 30 s at 72 °C. The reaction products were diluted, run on an ABI Prism 3100 Avant automated sequencer, and analyzed using GeneMapper software v6 (ABI). The primers for the polymorphic markers flanking the POT1 gene were TG1 (chr7:123,781,554), TG2 (chr7:124,091,551), AC1 (chr7:124,177,355), GT1 (chr7:124,189,252), GT2 (chr7:124,369,137), GT3 (chr7:124,428,420), GT5 (chr7:125,163,190), GT6 (chr7:125,288,073), AC2 (chr7:125,392,256), and AC3 (chr7:125,567,192). The forward primer for the variant was GGTTTGGTGTTTTGAAGTAAGCA, and the reverse primer was TTTCTGGGGAATGAAAGCAG.

## 3. Results

**Figure 1:**
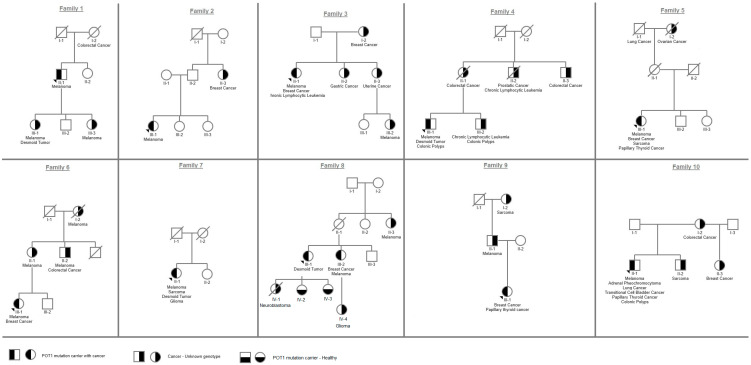
Pedigrees of all variant carriers and their family members included in the study.

The p.(I78T) germline missense variant in the *POT1* gene was detected in eleven probands (eight females, three males) from ten apparently unrelated families, all of AJ descent (Figure 1). Nine patients (82%) were diagnosed with melanoma between the ages of 25 and 63 years (Table 1). Seven of them (78%) reported recurrence of melanoma in different body parts, and most had undergone more than three resections at the time of the genetic consults. One patient (family 6, patient III-1) reported 10 recurrent resections, and one (family 4, patient 4-III-1) reported many additional resections of dermal nevi with high atypia. Tumor burden among carriers was high, with 30 primary malignancies (range 1–6; ages 25–67) (Table 1). Eight index patients reported a wide array of tumors. They included breast cancer in five (63% of all female carriers; ages 44, 44, 54, 51, and 67), sarcoma in two, desmoid tumors in three, and PTC in three. Other tumors included CLL, oligodendroglioma, neuroendocrine tumor, pheochromocytoma, lung cancer, urinary transitional cell carcinoma (TCC), colorectal cancer, prostate cancer, and adenomatous polyposis. One patient (family 10, patient 10-II-1), who was a heavy smoker, was first diagnosed with cancer at age 59 and had six different tumors, including multi-focal non-small-cell lung adenocarcinoma and TCC. The age range for the occurrence of the first tumor was 25–59 years. For two of them, melanoma was not the first tumor to occur.

Family history of various malignancies was reported in nine families (90%) (Figure 1, Table 1). The proband of family 1 reported two daughters diagnosed with malignant melanoma at ages 20 and 27 (patients III-1; III-3); one of them (patient III-1) also had a desmoid tumor at age 40. The proband of family 4 reported two relatives with CLL (patients II-2 and III-2), and the proband of family 6 reported three relatives with early-onset melanoma (patients I-2, II-1, and II-2). Proband III-1 from family 8 had a child who died from pediatric neuroblastoma. In six families (1, 3, 4, 6, 8, and 9) the age of first cancer reported was earlier in the younger generation; however, genotyping was only available for probands and not for the affected family members. Cascade testing was performed on two healthy carriers from family 8 (patients IV-2 and IV-3) who were found to be carriers for the familial variant at the age of 18 and 21 and were referred to clinical evaluation.

Other genes associated with the common tumors reported, especially melanoma, and the common malignancies of colorectal, breast, and prostate cancer, were included in all tests performed (Supplementary File S1). Only one patient (family 6, patient III-1) was found to carry an additional disease-causing variant, c.1273G>A p.Glu425Lys, in the MITF gene (NM_001354604.2), also known to be associated with melanoma. This patient reported a high recurrence rate of melanoma, having had 10 different resections over the years.

To gauge the allelic frequency of the p.(I78T) variant in the AJ population, a review of a local exome database was conducted. The database comprised 2721 exomes analyzed from 2018 to 2023 for various morbidities, mainly intellectual disability. The allelic frequency of the p.(I78T) variant across the whole database was 0.06% and was 0.25% among individuals identifying themselves as AJs. 

Haplotype analysis was conducted in six of the carriers of unrelated families. The 10 microsatellite markers that were tested represent the haplotype and are presented in Supplementary File S1. All the carriers tested were found to share the same haplotype.

## 4. Discussion

The (NM_015450.3) c.233T>C p.(Ile78Thr) variant is located in a conserved domain of POT1 that interacts with telomere DNA. Functional studies have shown that it alters the OB1 region, leading to the impairment of *POT1* single-stranded DNA binding and resulting in increased telomere length [11]. Assays of POT1 proteins and mRNA in lymphoblastoid cell lines derived from carriers of *POT1* p.(I78T) showed decreased expression and defective binding to telomere DNA, indicating that this missense variant has a loss-of-function effect. It has been shown that haploinsufficiency for *POT1* facilitates telomerase-dependent telomere elongation, and that long telomeres extend the replicative potential of cultured cells [11]. Telomere elongation is associated with telomere fragility, replication defects, and genomic instability. Indeed, carriers of *POT1* pathogenic variants were shown to have longer telomeres, and progenitors from *POT1* carriers displayed elevated somatic mutation burden compared to noncarriers [17].

Conflicting interpretations of pathogenicity are reported in ClinVar (variation ID# 475073); however, the functional support and the multiple prediction scores (e.g., SIFT = 0, REVEL = 0.69, and alphamissense 0.7842) suggest that it is deleterious. Bioinformatic analysis according to the guidelines for the classification of sequence variants of the ACMG [19] was carried out. PS4 criteria were used according to previously described patients and those described in our work, as elaborated in Table 2; PS3 criteria were used based on functional studies that have shown that the variant disrupts POT1–telomere binding and results in elongated telomeres as compared to controls [17]. PM1 criteria were used as the variant is located in the *POT1* domain, with a missense constraint value of 0.49, and other reported missense variants. For PM2, the variant frequency in the gnomAD v4 database is 0.0087%, while in the AJ sub-population the frequency is 0.02%. However, PM2 criteria can still be used, following the ACMG recommendations of downgrading it to a supporting level; PP3 criteria were used as computational prediction tools support a deleterious effect on the protein (REVEL 0.69, alphamissense 0.784). Taken together, based on the scoring of PS4 (4), PS3_moderate (2), PM1 (2), PM2_supporting (1), and PP3(1), the variant was classified as pathogenic. 

This variant has been mentioned previously (Table 2). All five previous publications describe the high occurrence of melanoma, and three report lymphoid malignancies among carriers; two have also described the occurrence of thyroid malignancy. The current publication describes a wider spectrum of solid malignancies apart from melanoma; we describe breast cancer in five of the eight female carriers, abdominal desmoid tumors in three carriers, colonic polyps in two carriers, and other tumors (Table 2).

Carriers in our cohort had high risk of recurrent melanoma (3 to 10), starting at the age of 25, earlier than the previously reported age of 31 [18]. In one first-degree non-genotyped family member, a melanoma had already appeared already at 20 years old. Interestingly, the sole patient found to be a “double carrier” of the *POT1* and *MITF* variants had an extremely high recurrence rate of melanoma (reporting 10 different resections over the years), possibly reflecting an additive/synergistic effect of the two variants.

CLL was diagnosed in one carrier and in two family members of another carrier. *POT1* was previously reported to be a candidate gene in lymphoid malignancies. No other genes are known to be associated with a definite risk of CLL. A recent study of 576 patients with CLL found a 3.3% prevalence rate of disease-causing germline and somatic loss-of-function variants in *POT1*. Others reported the poor prognostic implications of somatic and germline *POT1* variants in carriers with CLL, including shorter survival times and reduced response to treatments [23,24].

Desmoid tumors were reported in four families in our cohort, two located intra-abdominally and two within the abdominal wall. Tumors were recurrent and locally aggressive in all cases, appearing in different areas and requiring surgery. Disease onset occurred at ages 40, 41, 60, and 66. None of the patients had had previous abdominal trauma or surgery, both risk factors for abdominal desmoids.

Three of the patients with desmoid tumors underwent genetic testing and were found to carry the *POT1* variant. It is of note that one of these three carriers also had multiple adenomatous colonic polyps, raising the suspicion of APC-associated polyposis; however, neither him, nor the other two patients with desmoids, were found to carry a disease-causing variant in APC or in other polyposis-related genes.

Abdominal desmoids are rare fibroblastic tumors. They usually do not metastasize, but they are locally aggressive. Most are not associated with inherited conditions. The only gene reported so far to be associated with a definite risk for desmoids is APC (5–15%). In a small series of patients with desmoid tumors, disease-causing variants were identified in CHEK2, ERCC5, BLM, MSH6, and PALB2 [25]; however, none of these genes has been shown to be associated with increased risk of having desmoids. In the present study, desmoid tumors were reported in 40% of the evaluated families; a mesenteric desmoid tumor was reported recently in one *POT1* carrier [17]. To the best of our knowledge, these are the first reports of desmoid tumors among carriers of a disease-causing *POT1* variant. 

Three carriers (family 5, family 9, and family 10) were diagnosed with recurrent PTC at ages of 31, 41, and 63 years and were operated on more than once. Recently, *POT1* was suggested to be a candidate gene for familial non-medullary thyroid cancer based on the observation of a single family in which multiple members diagnosed with PTC were found to be carriers of the p.(V29L) variant in *POT1* [26]. None of these carriers were reported to have melanoma, CLL, or desmoid tumors. PTC is reported in two other families with the p.(I78T) variant (Table 2). The association between *POT1* and thyroid cancer remains provisional and justifies further research. 

DeBoy et al. [17] suggested occurrence of genetic anticipation, wherein offsprings of *POT1* disease-causing variant carriers had cancer that developed several decades earlier than that in their parents. Our data also suggest anticipation, based on the age of occurrence of the first tumor in families 1, 3, 4, 6, and 8. The proband of family 1 was diagnosed with melanoma at the age of 40, while her two daughters developed the disease at ages of 20 and 27; the proband of family 6 developed melanoma 10 years earlier than her mother and uncle; and the proband of family 8 had a child with neuroblastoma. However, as most family members have not been genotyped, this aspect should be further studied.

We did not look into the pathological or molecular features of the tumors reported. One study reported the common occurrence of spitzoid melanocytic neoplasms, characterized by large epithelioid melanocytes with abundant cytoplasm in *POT1* variant carriers, where 23 of the 30 melanomas studied had a spitzoid morphology involving at least 25% of the tumor [21]. Looking for unique features of *POT1*-associated tumors may provide insights regarding tumor behavior and, perhaps, clues for diagnosis.

All carriers in our study were AJs. The variant is very rare among healthy individuals, with allelic frequency in the gnomAD v.4.0.0 dataset of 0.0009%, detected in individuals from Ashkenazi and European descent. No homozygotes have been reported. Haplotype analysis revealed a shared haplotype, indicating that a common founder was likely in these families. We deduce that the carrier rate in AJs might be higher in patients with melanoma, particularly with early age of onset, more than one malignancy or significant family history. 

There are no published guidelines for the surveillance of individuals with a *POT1* tumor predisposition. It has been suggested that decisions regarding individual surveillance protocols should be based on the emerging phenotype spectrum of *POT1* variants and on the affected individual’s personal and family history. In addition, it has been recommended to employ screening similar to that used in LFS, due to the similarity to Li-Fraumeni syndrome [27]. In line with this suggestion, we recommend that carriers of *POT1* variants be referred for dermatological examination at least every 6 months beginning at the age of 18 years. It may be prudent to also refer carriers of the p.(I78T) variant to surveillance for thyroid, breast, and colon cancer, or to consider whole body MRI. On the basis of the present series, we advocate that individual surveillance protocols should be determined with consideration of both the evolving phenotypic spectrum and the personal and family history of the affected individuals and also take into consideration the variant involved. 

## 5. Conclusions

Here, we describe patients from ten unrelated families of AJ descent found to carry a disease-causing missense variant in the *POT1* gene, previously classified as VOUS. This is a founder variant with allelic frequency among AJs of 0.25%. This variant is associated with high tumor burden, risk for melanoma of earlier onset than previously reported, and an unprecedented link with various solid tumors, especially desmoid tumors, PTC, and breast cancer. This missense variant affects the OB1 region of the protein, resulting in increased telomeric length. We advocate testing for this variant in high-risk patients of Ashkenazi descent. In addition, the inclusion of *POT1* in germline panels for various types of cancer is warranted. Further research is needed to define the landscape of cancer types, establishing guidelines for surveillance, and deciphering the molecular characteristics of the resulting tumors and their possible varying responses to treatments.

## Figures and Tables

**Table 1:** Probands from ten unrelated families of Ashkenazi Jewish ancestry found to harbor the *POT1* I78T variant.

Patient Sign in Family Tree	Sex	Current Age (yrs)	Cancer (Age at Diagnosis)	Cancer in Non-Genotyped Family Members (Number of Individuals)
**1-II-1**	M	67	Melanoma (40)	Melanoma (2) Desmoid (1)
**2-III-1**	F	34	Melanoma (25)	Breast cancer (1)
**3-II-1**	F	68	Melanoma (58) CLL (64) Breast cancer (67)	Melanoma (1) Breast cancer (1) Uterine cancer (1) Gastric cancer (1)
**4-III-1**	M	52	Colonic polyps (25) Melanoma (35) Desmoid (41)	CLL (2) Colorectal cancer (1) Polyposis (1)
**5-III-1**	F	75	PTC (40) Soft tissue sarcoma (48) Melanoma (54) Breast cancer (54)	Ovarian Cancer (1)
**6-III-1**	F	51	Melanoma (35) Breast cancer (44)	Melanoma (3) Colorectal cancer (1)
**7-II-1**	F	68	Melanoma (45) Oligodendroglioma (60) Liposarcoma (65) Desmoid tumor (66)	
**8-III-1** **8-III-2**	F F	61 64	Desmoid tumor (60) Breast cancer (51) Melanoma (51)	Neuroblastoma (1) Glioma (1)
**9-III-1**	F	45	PTC (31) Breast cancer (44)	Melanoma (1)
**10-II-1**	M	67	Colonic polyps (59) Adrenal pheochromocytoma (59) Transitional cell carcinoma (60) Neuroendocrine tumor (60) Lung cancer (62) PTC (63) Melanoma (63)	Breast cancer (1) Colorectal cancer (1) Sarcoma (1)

**Abbreviations:** CLL: chronic lymphocytic leukemia, PTC: papillary thyroid cancer.

**Table 2:** Current and previous publications of p.(I78T) variant carriers.

1st Author, Year	Ancestry	Number of Carriers	Spectrum of Cancers
**Current publication**	Ashkenazi Jewish	Eleven carriers	■ Melanoma (9/11) ■ Breast cancer (5/8 women) ■ Desmoid (3/11) ■ Polyposis (2/11) ■ PTC (3/11) ■ Sarcoma (2/11) ■ CLL (1/11) ■ Glioma (1/11) ■ Pheochromocytoma (1/11) ■ Transitional cell carcinoma (1/11) ■ Neuroendocrine tumor (1) ■ Lung cancer (1/11)
**Wong, 2019** **[18].**	Jewish	Three families	■ Melanoma ■ Thyroid cancer ■ CLL ■ Cutaneous T-cell lymphoma
**Potrony, 2019** **[20].**	Spanish	One family	■ Melanoma ■ Thyroid cancer ■ Lung cancer
**Goldstein, 2023** **[21].**	European	One carrier	■ Melanoma
**Herrera-Muller** **[22].**	Ashkenazi Jewish	Sixteen carriers	■ Melanoma ■ Sarcoma ■ Prostatic ■ Kidney cancer
**Deboy, 2023** **[17].**	n/a	One family	■ Melanoma ■ B-cell lymphoma ■ T-cell lymphoma

**Abbreviations:** CLL: chronic lymphocytic leukemia, PTC: papillary thyroid cancer.

## Data Availability

The data presented in this study are available on request from the corresponding author. The data are not publicly available due to privacy and ethical issues.

## Supplementary Materials

The following supporting information can be downloaded at: https://www.mdpi.com/article/10.3390/genes15030355/s1, File S1: The list of tested genes, panel’s sequencing methods, and the genotyping technique of exome sequencing.

## Abbreviations

AJ	Ashkenazi Jewish
CLL	Chronic Lymphocytic Leukemia
CNV	Copy Number Variation
PCR	Polymerase Chain Reaction
POT1	Protection of Telomeres 1
PTC	Papillary Thyroid Cancer
TCC	Urinary Transitional Cell Carcinoma
VUS	Variant of Unknown Significance
