# Supplementary material for: A POT1 Founder Variant Associated with Early Onset Recurrent Melanoma and Various Solid Malignancies
Source: Genes (Basel). 2024 Mar 13;15(3):355. doi: 10.3390/genes15030355 (PMC10970179; doi:10.3390/genes15030355)
Supplement: Supplementary file 1 [file genes-15-00355-s001.zip › genes-2895719-supplementary.pdf]

Supplementary S1

List of genes included and sequencing methods, Clalit Genomic Center Hereditary Cancer Panel GC_28 (Clalit Genomic Center, Petach Tikva, Israel)

- **Genes tested**

AIP, ALK, ANKRD26, APC, ATM, AXIN2, BAP1, BARD1, BLM, BMPR1A, BRAF, BRCA1, BRCA2, BRIP1, BUB1B, CASR, CBL, CD70, CDC73, CDH1, CDK4, CDKN1B, CDKN1C, CDKN2A, CEBPA, CEP57, CHEK2, CTNNA1, CYLD, DDB2, DDX41, DICER1, DIS3L2, DKC1, DPYD, EFL1, EGFR, ELANE, EPCAM, ERCC1, ERCC2, ERCC3, ERCC4, ERCC5, ETV6, EXO1, EXT1, EXT2, EZH2, FAM111B, FANCA, FANCB, FANCC, FANCD2, FANCE, FANCF, FANCG, FANCI, FANCL, FANCM, FH, FLCN, GALNT12, GATA2, GPC3, GPR101, GREM1, HAVCR2, HNF1A, HOXB13, HRAS, IKZF1, ITK, KIF1B, KIT, KITLG, KRAS, LZTR1, MAP2K1, MAP2K2, MAX, MEN1, MET, MITF, MLH1, MLH3, MRE11, MSH2, MSH3, MSH6, MTAP, MUTYH, NBN, NF1, NF2, NRAS, NSD1, NSUN2, NTHL1, NUDT15, PALB2, PAX5, PDGFRA, PHOX2B, PMS1, PMS2, POLD1, POLE, POLH, POT1, PPM1D, PRF1, PRKAR1A, PRSS1, PTCH1, PTCH2, PTEN, PTPN11, PTPRJ, RAD50, RAD51C, RAD51D, RAF1, RASA2, RB1, RECQL, RECQL4, REST, RET, RHBDF2, RIT1, RPS20, RRAS, RUNX1, SAMD9, SAMD9L, SBDS, SDHA, SDHAF2, SDHB, SDHC, SDHD, SHOC2, SLX4, SMAD4, SMARCA4, SMARCB1, SMARCE1, SOS1, SOS2, SPRED1, SRP72, STK11, SUFU, TERC, TERT, TGFBR1, TINF2, TMEM127, TP53, TPMT, TRIP13, TSC1, TSC2, UGT1A1, VHL, WAS, WRN, WT1, XPA, XPC, XRCC2

- **Sequencing methods**

Sequencing was performed on an Illumina NovaSeq 6000 platform. The Illumina DNA prep with Enrichment (San Diego, CA, USA) was used with the IDT xGen Exome Research Panel v2 kit, producing paired-end sequence reads with an expected coverage above 80X. Resulting sequences were aligned against the human genome build GRCh37/UCSC hg19 and analyzed for sequence variants.

.

The analysis pipeline was independently validated for both single nucleotide variants and copy number variants. Performance of this assay is expected to fall within the same range as that of a validation test performed using a gold standard variant set ["Genome in a Bottle" (Coriell Institute, Camden, NJ)].

List of genes included and sequencing methods, Invitae Multi-Cancer Panel (Invitae COPREP, San Francisco, CA, USA)

- **Genes tested**

AIP, ALK, APC, ATM, AXIN2, BAP1, BARD1, BLM, BMPR1A, BRCA1, BRCA2, BRIP1, CASR, CDC73, CDH1, CDK4, CDKN1B, CDKN1C, CDKN2A, CEBPA, CHEK2, CTNNA1, DICER1, DIS3L2, EGFR, EPCAM, FH, FLCN, GATA2, GPC3, GREM1, HOXB13, HRAS, KIT, MAX, MEN1, MET, MITF, MLH1, MSH2, MSH3, MSH6, MUTYH, NBN, NF1, NF2, NTHL1, PALB2, PDGFRA, PHOX2B, PMS2, POLD1, POLE, POT1, PRKAR1A, PTCH1, PTEN, RAD50, RAD51C, RAD51D, RB1, RECQL4, RET, RUNX1, SDHA, SDHAF2, SDHB, SDHC, SDHD, SMAD4, SMARCA4, SMARCB1, SMARCE1, STK11, SUFU, TERC, TERT, TMEM127, TP53, TSC1, TSC2, VHL, WRN, WT1

- **Sequencing methods**

Genomic DNA obtained from the submitted sample was enriched for targeted regions using a hybridization-based protocol and sequenced using Illumina technology. Unless otherwise indicated, all targeted regions were sequenced with ≥50x depth or supplemented with additional analysis. Reads were aligned to a reference sequence (GRCh37), and sequence changes were identified and interpreted in the context of a single clinically relevant transcript, indicated below. Enrichment and analysis focused on the coding sequence of the indicated transcripts, 20bp of flanking intronic sequence, and other specific genomic regions demonstrated to be causative of disease at the time of assay design. Promoters, untranslated regions, and other non-coding regions were not otherwise interrogated. For some genes, only targeted loci were. Exonic deletions and duplications were called using an in-house algorithm that determines copy number at each target by comparing the read depth for each target in the proband sequence with both mean read-depth and read-depth distribution, obtained from a set of clinical samples. Markers across the X and Y chromosomes were analyzed for quality control purposes. Deviations from the expected sex chromosome complement that were detected may be included in the report in accordance with internal guidelines. The presence and location of reportable variants was confirmed according to stringent criteria established by Invitae (#05D2040778), as needed, using one of several validated orthogonal approaches (PubMed ID 30610921). The following analyses were performed if relevant to the requisition. For PMS2 exons 12-15, the reference genome was modified to force all sequence reads derived from PMS2 and the PMS2CL pseudogene to align to PMS2, and variant calling algorithms are modified to support an expectation of 4 alleles. If a rare SNP or indel variant was identified by this method, both PMS2 and the PMS2CL pseudogene were amplified by long-range PCR, and the location of the variant wass determined by Pacific Biosciences (PacBio) SMRT sequencing of the relevant exon in both long-range amplicons. If a CNV was identified, MLPA or MLPA-seq was run to confirm the variant. If confirmed, both PMS2 and PMS2CL were amplified by long-range PCR, and the identity of the fixed differences between PMS2 and PMS2CL were sequenced by PacBio from the long-range amplicon to disambiguate the location of the CNV. The technical component of confirmatory sequencing wqs performed by Invitae Corp (#05D2040778). For C9orf72 repeat expansion testing, hexanucleotide repeat units were detected by repeat-primed PCR (RP-PCR) with fluorescently labeled primers followed by capillary electrophoresis. Interpretation reference benign (normal range) was 31 repeat units. A second round of RP-PCR utilizing a non-overlapping set of primers was used to confirm the initial call in case of suspected allele sizes of 22 or more repeats. For RNA

analysis of the genes indicated in the Genes Analyzed table, complementary DNA was synthesized by reverse transcription from RNA derived from a blood specimen and enriched for specific gene sequences using capture hybridization. After high-throughput sequencing using Illumina technology, the output reads were aligned to a reference sequence (genome build GRCh37; custom derivative of the RefSeq transcriptome) to identify the locations of exon junctions through the detection of split reads. The relative usage of exon junctions in a test specimen was assessed quantitatively and compared to the usage seen in control specimens. Abnormal exon junction usage was evaluated as evidence in the Sherloc variant interpretation framework. If an abnormal splicing pattern was predicted based on a DNA variant outside the typical reportable range, as described above, the presence of the variant was confirmed by targeted DNA sequencing. RNA sequencing was performed by Invitae Corp.(#05D2094793). The technical component of fibroblast cell-culturing and gDNA extraction from skin punch biopsy was performed by Invitae Corp. (#05D1052995).

List of genes included and sequencing methods, Blueprint Comprehensive Hereditary Cancer Panel (Blueprint Genetics, Espoo, Finland)

- **Genes tested**

AIP, ALK, ANKRD26, APC, ATM, AXIN2, BAP1, BARD1, BLM, BMPR1A, BRAF, BRCA1, BRCA2, BRIP1, BUB1B, CBL, CD70, CDC73, CDH1, CDK4, CDKN1B, CDKN1C, CDKN2A, CEBPA, CEP57, CHEK2, CTNNA1, CYLD, DDB2, DDX41, DICER1, DIS3L2, DKC1, EFL1, EGFR, ELANE, EPCAM, ERCC1, ERCC2, ERCC3, ERCC4, ERCC5, ETV6, EXO1, EXT1, EXT2, EZH2, FAM111B, FANCA, FANCB, FANCC, FANCD2, FANCE, FANCF, FANCG, FANCI, FANCL, FANCM, FH, FLCN, GALNT12, GATA2, GPC3, GPR101, GREM1, HAVCR2, HNF1A, HOXB13, HRAS, IKZF1, KIF1B, KIT, KITLG, KRAS, LZTR1, MAP2K1, MAP2K2, MAX, MEN1, MET, MITF, MLH1, MLH3, MRE11A, MSH2, MSH3, MSH6, MUTYH, NBN, NF1, NF2, NRAS, NSD1, NSUN2, NTHL1, PALB2, PAX5, PDGFRA, PHOX2B, PMS1, PMS2, POLD1, POLE, POLH, POT1, PPM1D, PRF1, PRKAR1A, PTCH1, PTEN, PTPN11, RAD50, RAD51C, RAD51D, RAF1, RASA2, RB1, RECQL, RECQL4, REST, RET, RHBDF2, RIT1, RPS20, RRAS, RUNX1, SAMD9, SAMD9L, SBDS, SDHA, SDHAF2, SDHB, SDHC, SDHD, SHOC2, SLX4, SMAD4, SMARCA4, SMARCB1, SMARCE1, SOS1, SOS2, SPRED1, SRP72, STK11, SUFU, TERC, TERT, TINF2, TMEM127, TP53, TRIP13, TSC1, TSC2, VHL, WRN, WT1, XPA, XPC and XRCC2.

- **Sequencing Methods**

Laboratory process: When required, the total genomic DNA was extracted from the biological sample using the bead-based method. DNA quality and quantity were assessed using electrophoretic methods at Blueprint Genetics, and the qualified genomic DNA sample was then randomly fragmented using non-contact, isothermal sonochemistry processing. The sequencing library was prepared by ligating sequencing adapters to both ends of DNA fragments. Sequencing libraries were size-selected with the bead-based method to ensure optimal template size and amplified by polymerase chain reaction (PCR). Regions of interest (exons and intronic targets) were targeted using the hybridization-based target capture method. The quality of the completed sequencing library was controlled by ensuring the correct template size and quantity and eliminating the presence of leftover primers and adapter-adapter dimers. Ready sequencing libraries that passed quality control were pair-end sequenced using the Illumina's sequencing-by-synthesis method (150 by 150 bases). The sequencing instrument with Illumina proprietary software was applied for primary data analysis ,converting images into base calls and associated quality scores, generating CBCL files as the final output.These steps were performed at Blueprint Genetics. Bioinformatics and quality control: Base-called raw sequencing data were transformed into FASTQ format using Illumina software (bcl2fastq). Sequence reads of each sample were mapped to the human reference genome (GRCh37/hg19). Burrows-Wheeler Aligner (BWA-MEM) software was used for read alignment. Duplicate read marking, local realignment around indels, base quality score recalibration and variant calling were performed using GATK algorithms (Sentieon) for nDNA. Variant data for was annotated using a collection of tools (VcfAnno and VEP) with a variety of public variant databases, including but not limited to gnomAD, ClinVar, and HGMD. Median sequencing depth and coverage across the target regions for the tested sample were calculated on the basis of MQ0 aligned reads. The sequencing run included in-process reference sample(s) for quality control, which passed our thresholds for sensitivity

and specificity. The sample was subjected to thorough quality control measures including assessments for contamination and sample mix-up. Copy number variations (CNVs), defined as single exon or larger deletions or duplications (Del/Dups), were detected from the sequence analysis data using a proprietary bioinformatics pipeline. The difference between observed and expected sequencing depth at the targeted genomic regions was calculated, and regions were divided into segments with variable DNA copy numbers. The expected sequencing depth was obtained by using other samples processed in the same sequence analysis as a guiding reference. The sequence data were adjusted to account for the effects of varying guanine and cytosine content. Bioinformatics and quality control processes were performed by Blueprint Genetics. Interpretation: The clinical interpretation team assessed the pathogenicity of the identified variants by evaluating the information in the patient requisition, reviewing the relevant scientific literature, and manually inspecting the sequencing data if needed. All available evidence of the identified variants was compared to classification criteria. Reporting was carried out using HGNC-approved gene nomenclature and mutation nomenclature following the HGVS guidelines. Likely benign and benign variants were not reported. The interpretation was performed at Blueprint Genetics. Variant classification: Our variant classification follows the Blueprint Genetics Variant Classification Schemes modified from the 2015 ACMG guideline. Minor modifications were made to increase reproducibility of the variant classification and improve the clinical validity of the report. The classification and interpretation of the variant(s) identified reflect the current state of Blueprint Genetics' understanding at the time of this report. Variant classification and interpretation are subject to professional judgment, and may change for a variety of reasons, including but not limited to, updates in classification guidelines and availability of additional scientific and clinical information. This test result should be used in conjunction with the health care provider's clinical evaluation. Inquiry regarding potential changes to the classification of the variant is strongly recommended prior to making any future clinical decision. For questions regarding variant classification updates, please contact support@blueprintgenetics.com Databases: The pathogenicity potential of the identified variants was assessed by considering the predicted consequence of the change, the degree of evolutionary conservation, as well as the number of reference population databases and mutation databases such as, but not limited to, gnomAD, ClinVar, HGMD Professional, and Alamut Visual. In addition, the clinical relevance of any identified CNVs was evaluated by reviewing the relevant literature and databases such as Database of Genomic Variants and DECIPHER. For interpretation of mtDNA variants, specific databases were used, including for example,. Mitomap, HmtVar, and 1000G.Confirmation of sequence alterations: Sequence variants classified as pathogenic, likely pathogenic, and variants of uncertain significance (VUS) were confirmed using bi-directional Sanger sequencing when they did not meet our stringent NGS quality metrics for a true positive call. In addition, prenatal cases with diagnostic findings were confirmed.The confirmation of sequence alterations was performed at Blueprint Genetics. Confirmation of copy number variants: CNVs (deletions/duplications) were confirmed using a digital PCR assay if they covered less than 10 exons (heterozygous) or less than 3 exons (homo/hemizygous) or if they were not confirmed at least three times previously at our laboratory. Furthermore, CNVs of any size were not confirmed when the breakpoints of the call could be determined.The confirmation of copy number variants was performed at Blueprint Genetics. Analytic validation: The detection performance of this panel is expected to be in the same range as our high-quality, clinical grade NGS sequencing assay used to generate the panel data (nuclear DNA: sensitivity for SNVs 99.89%, indels 1-50 bps 99.2%, one-exon deletion 100% and five exons CNV 98.7%, and specificity >99.9% for most

variant types). It does not detect very low level mosaicism as a variant with minor allele fraction of 14.6% can be detected in 90% of the cases. Detection performance for mtDNA variants (analytic and clinical validation): sensitivity for SNVs and INDELs 100.0% (10-100% heteroplasmy level), 94.7% (5-10% heteroplasmy level), 87.3% (<5% heteroplasmy level) and for gross deletions 100.0%. Specificity is >99.9% for all.

Sequencing Methods of Whole Exome Sequencing, CeGat Laboratory (CeGaT GmbH, Tübingen, Germany**)**

Exome sequencing was done at CeGat laboratory). Targeted capture of protein-coding regions was performed using the Twist Human Core Exome Plus Kit (Twist Bioscience, San Francisco, CA, USA). Paired-end libraries were prepared from captured fragments and sequenced on the Illumina NovaSeq 6000 platform (Illumina, San Diego, CA, USA). Demultiplexing of the sequencing reads was performed with Illumina bcl2fastq (2.19). Adapters were trimmed with Skewer (version 0.2.2) (Jiang et al. 2014). Quality trimming of the reads has not been performed. The quality of FASTQ files was analyzed with FastQC (version 0.11.5-cegat) (Andrews 2010). Plots were created using ggplot2 (Wickham 2009) in R (R Core Team 2015).

FASTQ files, along with information on phenotypes using human phenotype ontology (HPO) terms and family structure were uploaded into Emedgene's HIPAA-compliant platform (Emedgene Technologies, Ltd, Mazor, Israel) and analyzed as described previously. (PMID: 30377382, 2019). Briefly, parameters used for variant interpretation included mapping quality ≥45 and depth ≥10, population frequency (1% or 5% for dominant or recessive inheritance, respectively), and variant severity. Analysis of copy number variants was not performed. Variants were classified according to the criteria of the American College of Medical Genetics (ACMG) (PMID: 25741868, 2015).

Microsatellite analysis - Founder haplotype colored in blue							
hg19	Marker	1	2	3	4	5	6
123,781,554	TG1	223/232	232/234	221/232	232	221/232	232/234
124,091,551	TG2	210	210/216	208/210	210/216	210	210
124,177,355	AC1	169/173	169	169/171	167/169	169	169/175
124,189,252	GT1	248/257	244/257	257/261	244/257	252/257	257/259
124,369,137	GT2	**232**/234	**232**	219/**232**	**232**	228/**232**	**232**
124,428,420	GT3	**244**/246	**244**	**244**/246	**244**	**244**	**244**
MUT c.233T>C	T\C	T\C	T\C	T\C	T\C	T\C	T\C
125,163,190	TG5	**259**/263	**259**	254/**259**	252/**259**	**259**/263	-
125,288,073	TG6	268	261/268	261/268	266/268	268	263/268
125,392,256	AC2	212/215	212	212	212	212	212/215
125,567,192	AC3	212/221	212/219	212/223	212/219	212/222	212/219
